# The neurostructural bases of empathy: morphometric evidence for a multicomponential approach

**DOI:** 10.3389/fpsyt.2025.1544632

**Published:** 2025-04-10

**Authors:** Maria Arioli, Leonardo Rassouli Baghi, Zaira Cattaneo, Nicola Canessa

**Affiliations:** ^1^ Department of Human and Social Sciences, University of Bergamo, Bergamo, Italy; ^2^ IUSS Cognitive Neuroscience (ICoN) Center, Scuola Universitaria Superiore IUSS, Pavia, Italy; ^3^ Istituti Clinici Scientifici Maugeri IRCCS, Cognitive Neuroscience Laboratory of Pavia Institute, Pavia, Italy

**Keywords:** empathy, brain morphometry, personal distress, empathic concern, sex differences, intervention, amygdala, insula

## Abstract

The neural bases of individual differences in empathy subcomponents are still debated. We employed brain morphometry to investigate the neurostructural bases of individual and sex differences in specific empathy facets in 124 healthy individuals who completed the Balanced-Emotional-Empathy-Scale (BEES), and both the emotional/cognitive and self/other-oriented empathy subscales of the Interpersonal-Reactivity-Index (IRI). Univariate and multivariate morphometric analyses highlighted, respectively, voxels/clusters and whole structural networks where grey-matter volume reflected specific empathy subscores. Such morphometric properties were significantly related to individual differences in *emotional empathy*, while no evidence was found for structural networks underlying *cognitive empathy*. Personal distress correlated with grey-matter volume in the right insula and amygdala, likely mediating an affective sharing self-perceived as disturbing. Instead, empathic concern was associated with the medial precuneus and sensorimotor/inferior parietal cortex, possibly enabling empathic comprehension and prosocial behaviour mediated by attentional shift towards others. Female participants displayed larger grey-matter volume than male ones, related to higher emotional empathy, in limbic structures including amygdala and insula. These results ground multicomponential empathy models in specific neurostructural networks, representing a reference for future studies of empathic processing in health and disease.

## 1 Introduction

Many routes in neuroscience cross the notion of empathy, i.e., the ability to recognize and share others’ feelings (1, 2), by addressing its neural correlates (3) and/or its breakdown in neuropsychiatric conditions (4). Empathy is considered a multifaceted concept, including emotional (feeling another’s emotional situation) and cognitive (or mentalizing, i.e., making inferences on another’s mental states; 5, 6) processes, alongside self-oriented (inner simulation) or other-oriented (third-person focus) reactions (7, 8). These components are considered to involve specialized brain systems.

Cognitive empathy has been associated with the medial prefrontal cortex, temporoparietal junction (TPJ), and temporal pole (5, 9). Instead, specific neural correlates underpin the distinct emotional empathic reactions associated with the apprehension of another’s state (i.e., personal distress, an aversive self-focused reaction) and empathic concern (or compassion, an other-oriented response of concern) (10, 11). The former is associated with the concept of a “shared” perception-action mechanism, mapping another’s sensory/affective states on corresponding inner neural representations (12, 13), recruiting structures commonly activated both by first- and third-person sensorimotor events (i.e., fronto-parietal areas underlying motor mirroring; 14) and affective experiences such as pain (i.e., insula and anterior cingulate cortex (ACC); 15–17). Instead, empathic concern appears to engage the ventral striatum and medial orbitofrontal cortex, supporting feelings of warmth and prosocial motivations (18). Despite a general agreement on this taxonomy of empathy components, their mutual associations remain debated.

Different views suggest either that emotional empathy includes personal distress and empathic concern (e.g., 7, 19, 20), that empathic concern represents the unique possible emotional empathic reaction (since *empathy needs to be other-oriented*, e.g., 21, 22), or that empathic concern represents a unique construct distinct from both cognitive empathy and personal distress of emotional empathy (e.g., 8, 23–25). Regardless of their possible mutual relationships, empathic concern is generally considered a more complex, top-down (24) and high-level empathic reaction compared with personal distress (8). Moreover, empathic concern is considered to share some features with cognitive empathy (26, 27; see 25 for different models).

The interpersonal reactivity index (IRI; 28) is a widely used questionnaire for studying these empathic components in clinical (29) and research (30) settings. The IRI results in four subscores involving emotional vs. cognitive processes and self-oriented vs. other-oriented reactions: personal distress and fantasy (self-oriented emotional and cognitive empathy, respectively), as well as empathic concern and perspective-taking (other-oriented emotional and cognitive empathy, respectively).

Alongside functional neuroimaging, brain morphometry provides valuable insights into the neural bases of empathy (31–35), while also tracking individual differences in social variables such as network size (36), sense of humour (37) and harm aversion (38). Moreover, quicker and easier data collection makes this approach a preferable alternative to activation paradigms when studying clinical populations. Previous studies have, however, provided inconclusive evidence on the *neurostructural bases* of the key empathy components. Only few studies used univariate Voxel-Based-Morphometry (VBM) to investigate a possible relationship between whole-brain regional grey matter (GM) density and IRI subscores. These studies reported both positive and negative correlations between different IRI subscores and GM density in the insula and ACC (39), while decreased volume in the bilateral anterior insula was also associated with increased fantasy and empathic concern (32) alongside personal distress (40; but see 41). These inconsistencies might reflect differences across studies concerning methodological aspects (e.g., sample size and statistical threshold), participants’ characteristics (e.g., sex distribution and cultural context), or primary aims (e.g., focus on a single dimension vs. specific neural correlates). Nevertheless, this complex pattern highlights the need of further inquiry on the neural precursors of empathic dispositions, including the well-known sex differences in empathy (42, 43). Despite consistent evidence of different brain responses - across female and male participants - in tasks tapping social cognition and empathy (44), morphometric sex differences have been only reported for personal distress (40). Moreover, better insights into the neurostructural bases of different empathy facets might come from analytic approaches other than mass-univariate VBM, and particularly by multivariate approaches such as Source-Based-Morphometry (SBM; 45). By pooling information across voxels to identify grouped regions showing similar inter-subject covariation (46), this approach might unveil structural networks reflecting individual differences in distinct empathy facets.

Unlike previous studies, we therefore used both univariate (VBM) and multivariate (SBM) morphometric analyses to investigate the relationship between GM volume (GMV) and individual differences (including sex effects) in specific empathy facets in 124 healthy young individuals. These two approaches highlighted, respectively, voxels/clusters and networks where GMV reflected individual differences on the Balanced-Emotional-Empathy-Scale (BEES; 47) and IRI subscales, thereby providing a comprehensive overview of the neuro-structural bases of empathy. We predicted to observe a relationship between personal distress and GMV in the insula (39; but see 40), while empathic concern might involve structures supporting prosocial behaviour (24), with an expected sex-related modulation (48) particularly involving the emotional empathy component (42, 44).

## 2 Methods

### 2.1 Participants

The experimental sample included 124 right-handed healthy young individuals (64 females; mean age=24 years, standard deviation [SD]=3.34), with no significant age difference between females (mean=23.63 years, SD=3.24) and males (mean=24.39 years, SD=3.43) (*t*(122)=1.27, *p*=0.20). All subjects reported no history of psychiatric or neurological disorders, nor of drug/substance use, and no current use of any psychoactive medications. They all gave their written informed consent to the experimental procedure, which had been approved by the local Ethics Committee.

### 2.2 Experimental procedure

Participants completed the Italian translation of the BEES (49) and IRI (50).

The BEES measures one’s vicarious experience of another’s emotional experiences (51), through 30 items measured on a nine-point Likert scale ranging from -4 (“It does not describe me at all”) to 4 (“It describes me at all”).

The IRI includes four subscales for the assessment of self-oriented and other-oriented measures of both emotional and cognitive empathy: “Personal distress” (tendency to experience self-oriented distress in response to others’ distress), “Empathic concern” (tendency to experience feelings of concern or compassion for unfortunate others), “Fantasy” (tendency to imaginatively transpose oneself into fictional situations) and “Perspective-taking” (tendency to spontaneously adopt another person’s point of view). Each subscale includes seven items, measured on a five-point Likert scale ranging from 0 (“It does not describe me well”) to 4 (“It describes me very well”). Although the model structure underlying the IRI remains controversial (52), following Davis’s original version and studies on Italian populations (53–55) we computed subscores according to the four-factor structure.

Participants completed the above questionnaires in counterbalanced order before MRI sessions. No missing values were recorded.

### 2.3 MRI-data acquisition and spatial pre-processing

T1-weighted brain scans (152 slices, slice thickness=1mm, in-plane resolution=1mm x 1mm) were acquired with a 3 Tesla General Electrics scanner (MR750 Discovery, GE Healthcare), using a 16-channels head coil. Image spatial pre-processing was performed with SPM12 and the CAT12 (https://neuro-jena.github.io/cat12-help/) toolbox. The pre-processing included (a) bias correction of intensity non-uniformities; (b) creation of an *ad-hoc* template based on the T1-weighted images of 308 age- and sex-matched healthy individuals; (c) spatial normalization of all 124 images to such template using the DARTEL toolbox (56); (d) extraction of GM and white-matter (WM) components from the normalized images; (e) multiplication of the GM segments by the non-linear components derived from the normalization matrix to perform volumetric analyses on “modulated” GM volumes; (f) smoothing (8 mm isotropic gaussian FWHM kernel) of the GM images.

### 2.4 VBM statistical analyses

We first employed two-sample t-tests to investigate sex differences in GMV, while controlling for the potential effect of global GMV. We then assessed a relationship with specific empathy measures, via multiple regression models to investigate the regions where GMV was positively related either to the BEES score or to a specific IRI emotional/cognitive subscore while controlling for participants’ age. To test the specificity of results for single IRI subscores, we included in a same model both the two emotional (or cognitive) empathy subscores (e.g., to test for personal distress while controlling for empathic concern). We then assessed sex effects on these relationships, by searching for voxels in which the regression slope was significantly different across females and males.

To prevent voxel misclassification on the GM-WM border, we set the absolute GM threshold at 0.15. The resulting statistical maps were thresholded at *p*<0.05, cluster-level corrected with topological False Discovery Rate (FDR) correction (57) (forming threshold=0.005). We used the Anatomy-Toolbox v2.2c (58) to localize the regions showing significant results.

### 2.5 SBM pre-processing

SBM employs multivariate spatial Independent Component Analysis (ICA) to decompose GM images into maximally independent spatial sources representing “natural structural networks” (46). The expression of such patterns in single participants is quantified by a “loading coefficient” that can be modeled in statistical analyses to investigate group differences or a relationship with variables of interest. SBM entails image pre-processing (identical to that performed for VBM), ICA, and statistical analysis. We used the GIFT toolbox (http://mialab.mrn.org/software/; 59) to perform ICA through a neural network algorithm (Infomax) that attempts to minimize the mutual information of the network outputs to identify naturally grouping and maximally independent sources (60). ICA was repeated 250 times in Icasso (http://research.ics.aalto.fi/ica/icasso/) and resulting components were clustered to ensure the reliability of results, which is quantified through a quality index (Iq) ranging from 0 to 1 and reflecting the difference between intra-cluster and extra-cluster similarity (61). All the 34 independent components (ICs) extracted from the GM images were associated with an Iq>0.8, indicating a highly stable ICA decomposition (62). Based on visual inspection, we excluded 9 components including potentially artefactual sources (e.g. extending into white matter or ventricles). We obtained anatomical labels of clusters using the Anatomy-Toolbox (v2.2c) (58).

### 2.6 SBM statistical analysis

For each of the 25 retained components we first used two-sample t-tests to compare the loading coefficients across female and male participants. We considered as female- or male-dominant those components surviving a statistical threshold of *p*<0.05 FDR corrected (63). For all components, we then assessed a positive relationship between individual empathy (sub)scores and individual loading coefficients. We first assessed the correlation between loading coefficients and single subscores, using a FDR-corrected *p*<0.05 threshold. This step highlighted significant effects of “emotional” empathy (BEES, Empathic concern and Personal distress), and no significant effect of “cognitive” empathy (either Fantasy or Perspective-taking). We then ran multiple regression models to assess whether the single empathy (sub)scores (dependent variable) are significantly predicted by the loading coefficients of the retained components. Since some loading coefficients were significantly cross-correlated, we used a maximum variance inflation factor (VIF) of 4 to quantify the severity of their multicollinearity. We obtained anatomical labels of clusters within each component using the Anatomy-Toolbox v2.2c (58).

## 3 Results

### 3.1 Empathy scores

**Figure 1:**
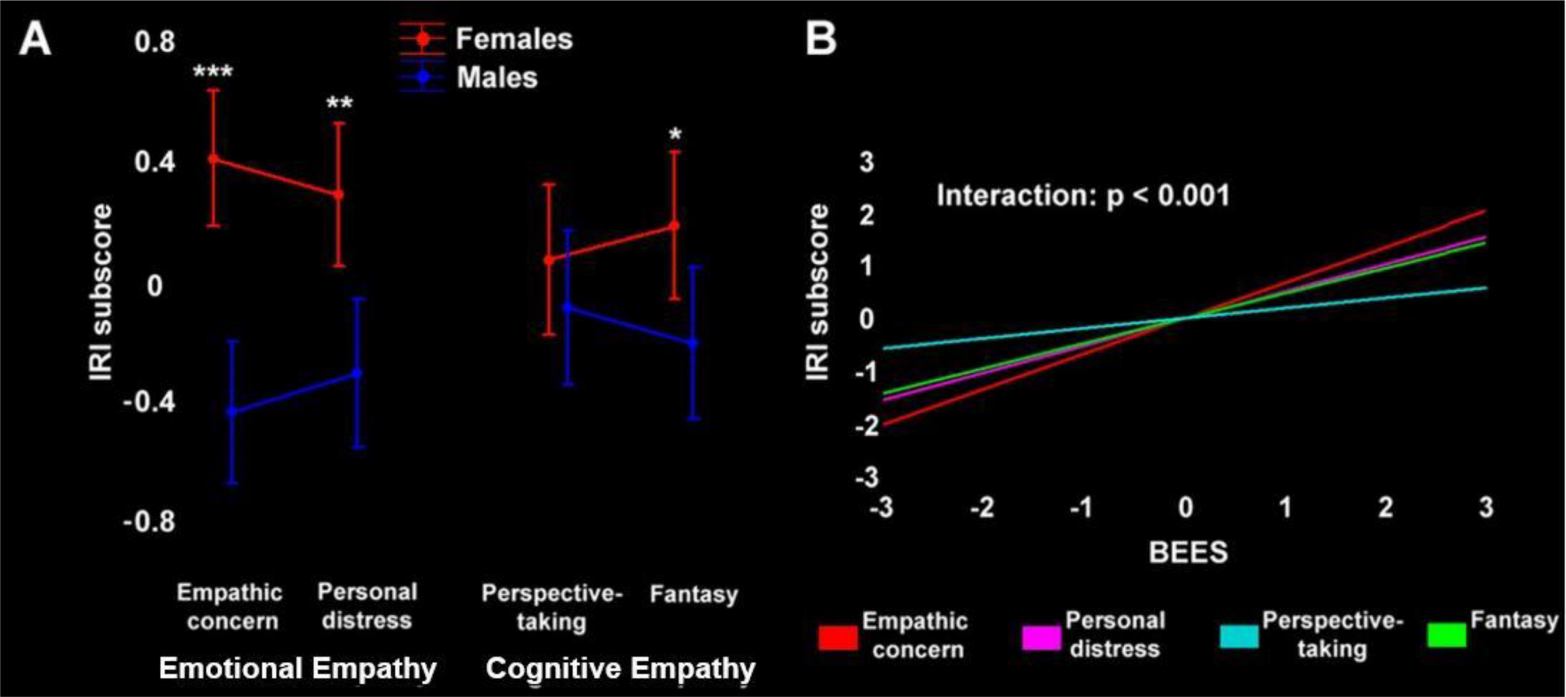
**(A)** Emotional and cognitive empathy (IRI subscores) in female (red) and male (blue) participants. The asterisks depict the statistical significance of *post-hoc* comparisons (***p<0.0001, **p<0.001, *p<0.05). **(B)** Statistical model assessing the correlation between BEES score and single IRI subscores, confirming the distinctiveness of Perspective-taking (*p*<0.001). To better convey the relationship between BEES and IRI (sub)scores and their modulation by sex, both panels depict their standardized values (the original values are reported in **Supplementary Table S1**).

BEES and all IRI scores were normally distributed (Kolmogorov-Smirnov, p>0.2). We observed significant sex effects, with females displaying higher empathy scores at the BEES, IRI global score and all IRI subscores except for perspective-taking (**Supplementary Table S1**). We tested an interaction between sex and IRI scores using a 2x2x2 ANOVA, with factors “sex”, “empathy type” (emotional vs. cognitive) and “empathy target” (other-oriented vs. self-oriented). While there was no significant three-way interaction, results highlighted a significant 2x2 interaction (*F*(1,122)=8.51, *p*=0.004) between “sex” and “empathy type”, with larger sex differences in emotional than cognitive empathy (**Figure 1A**), and no significant interaction between “sex” and “empathy target” (*F*(1, 122) = 0.003, *p*= 0.956).

We observed a strong positive correlation between BEES and IRI global score, that was largely driven by IRI emotional empathy. Indeed, we found significant strong correlations between BEES score and IRI Personal distress, Empathic concern and Fantasy subscores (all *p*<0.0001), and a weak correlation with Perspective-taking (**Supplementary Table S2**). A “separate slopes” model confirmed that the correlation between BEES and IRI was significantly different across specific subscores (*F*(3)=7, *p*<0.001), with *post-hoc* comparisons confirming that Perspective-taking is qualitatively distinct from the other IRI subscales (*p*<0.05) (**Figure 1B**). We found the same pattern of correlations also when assessing separately female and male participants.

There was no significant correlation between age and BEES, IRI global score or any of the IRI subscores. Moreover, the separate assessment of female and male participants highlighted no significant correlation between age and any empathy scores.

### 3.2 VBM results: sex differences in GMV

**Figure 2:**
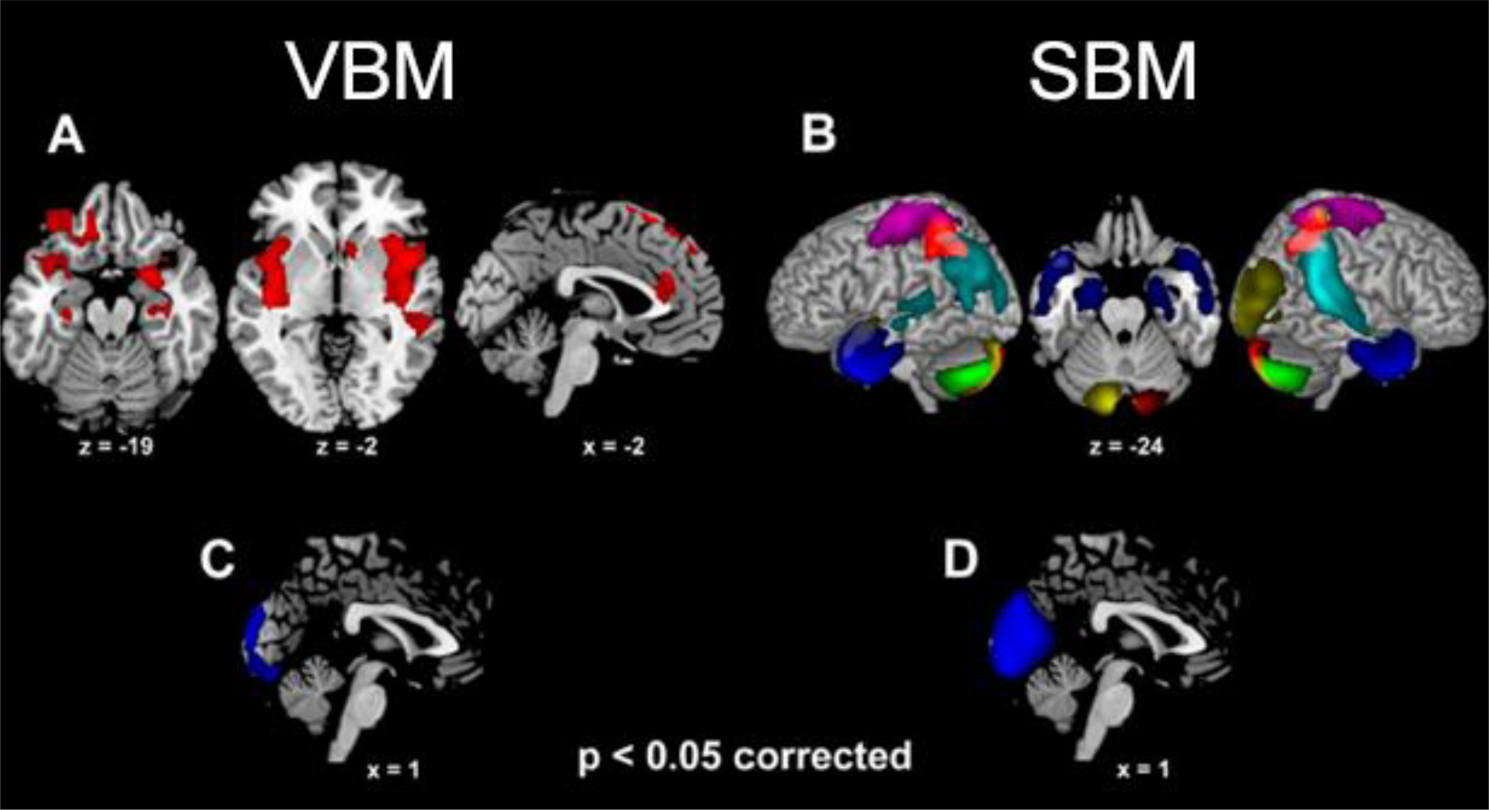
The clusters (VBM analysis) and structural networks (SBM analysis) showing larger grey matter volume in females than males [**(A, B)**, respectively] as well as in males than females [**(C, D)**, respectively] (*p*<0.05 corrected for multiple comparisons). For SBM, different colors depict different independent components (i.e., “natural structural networks”; see 3.4).

When controlling for global GMV, sex comparisons highlighted larger GMV in females, compared with males, in several limbic clusters (**Supplementary Table S3a**; **Figure 2A**). These included a left orbitofrontal cluster, extending from the inferior frontal gyrus (pars opercularis and orbitalis) to the middle and superior orbital gyri, as well as the amygdala, hippocampus, temporal pole and insula (from posterior to anterior sectors) bilaterally. Females displayed larger GMV also in the left sensorimotor cortex (encompassing postcentral and precentral gyri), medial superior frontal gyrus and dorsal ACC. In the reverse comparison, males displayed larger GMV than females in the medial occipital cortex (calcarine gyrus and cuneus) (**Supplementary Table S3b**; **Figure 2C**).

### 3.3 VBM results: correlation with BEES and IRI (sub)scores

**Figure 3:**
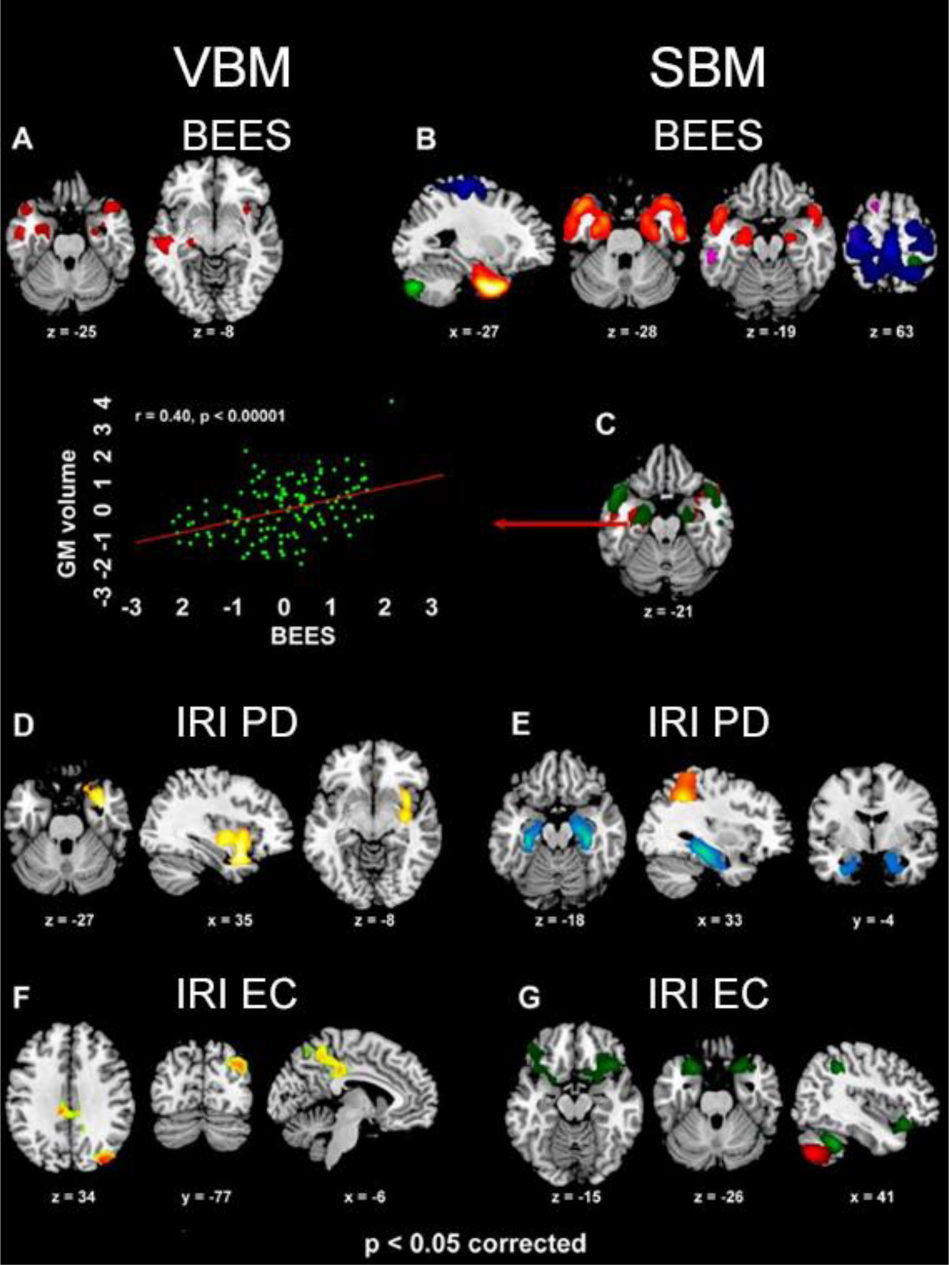
The clusters (VBM analyses) and structural networks (SBM analyses) where GMV was positively related with BEES [**(A, B)**, respectively], IRI Personal distress [**(D, E)** respectively] and IRI Empathic concern [**(F, G)** respectively] scores (*p*<0.05 corrected for multiple comparisons). In SBM results, BEES score reflects GMV in components 17 (violet), 18 (red-yellow), 19 (green) and 21 (blue), while Personal distress (PD) is associated with components 5 (light blue) and 31 (orange), and Empathic concern (EC) with components 19 (red) and 32 (green). The overlap between VBM and SBM neurostructural correlates of emotional empathy (BEES) in the bilateral amygdala and temporal pole is also shown **(C)**, along with a scatterplot depicting the significant correlation (*r*=0.40, *p*<0.0001) between GMV in the left amygdala and BEES score (standardized values).

The BEES score was positively correlated with GMV in the amygdala, hippocampal cortex and temporal pole bilaterally, alongside the right ventral anterior insula and left inferior-middle temporal cortex (**Supplementary Table S4a**; **Figures 3A, C**).

**Figure 4:**
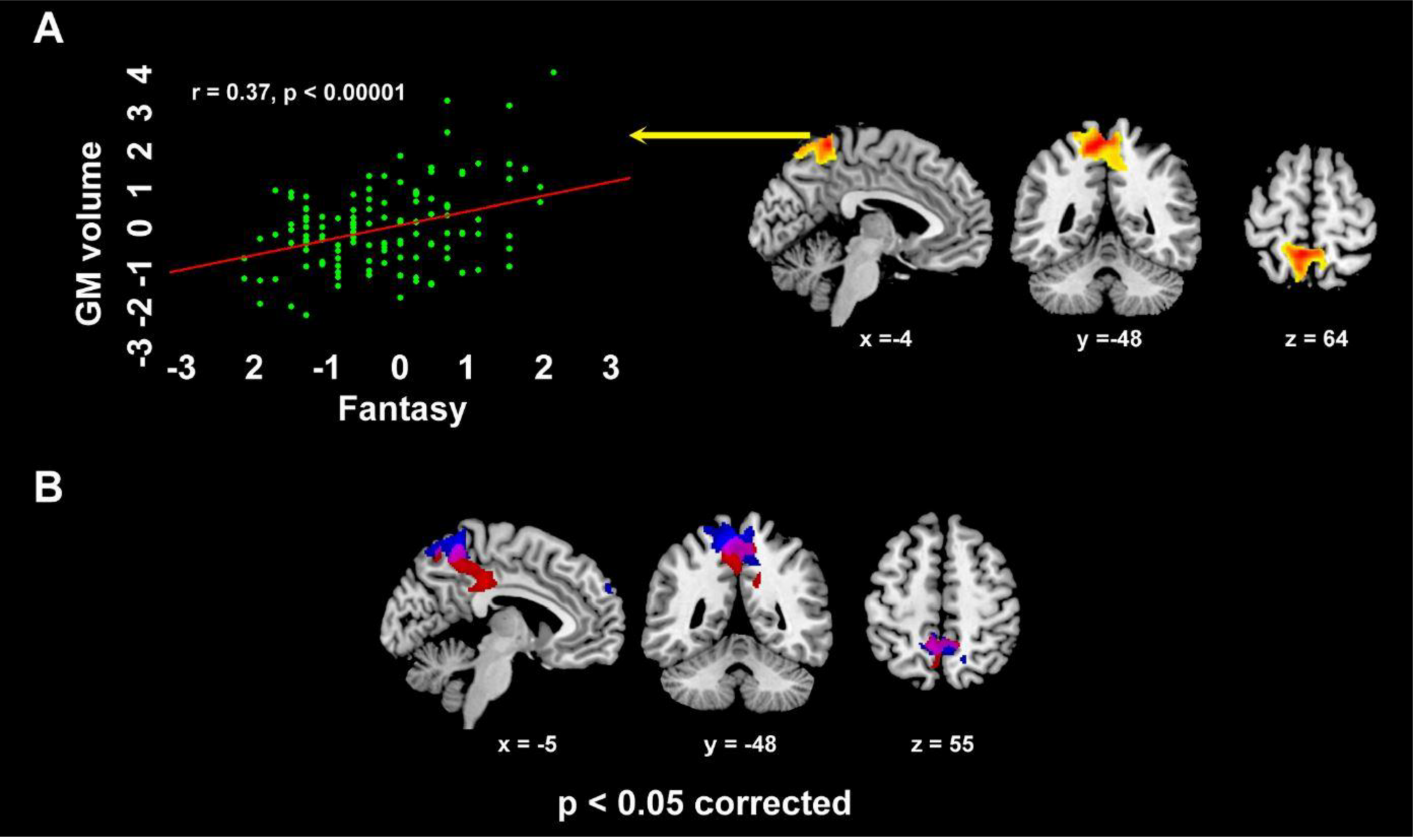
The dorsal medial parietal cluster where, in VBM analyses, GMV was positively related with IRI Fantasy (*r*=0.37, *p*<0.0001; **A**), alongside its overlap with the cluster - encompassing middle cingulate cortex and medial precuneus - where such correlation involved Empathic concern (violet color; **B**) (*p*<0.05 corrected for multiple comparisons).

IRI Personal distress reflected in increased GMV in a limbic cluster encompassing the right parahippocampal gyrus, amygdala, temporal pole, pars orbitalis of the inferior frontal gyrus, as well as ventral insula (from posterior to anterior sectors) (**Supplementary Table S4b**; **Figure 3D**. Empathic concern was specifically related with GMV in a medial parietal cluster encompassing middle cingulate cortex and medial precuneus (**Supplementary Table S4c**; **Figure 3F**), alongside the right middle occipital and angular gyri. Fantasy subscore was positively associated with GMV in the dorsal medial precuneus, extending into the superior parietal lobule bilaterally (**Supplementary Table S4d**; **Figure 4A**).

A conjunction-analysis confirmed the common involvement of the medial precuneus (-9 -49 57; p=0.048 corrected) both in Empathic concern (left: -9 -49 54; right: 10 -48 55) and Fantasy (left: -9 -49 -61; right: 3 -52 54) (**Figure 4B**). Instead, we did not observe significant correlations between GMV and Perspective-taking.

None of the above analyses displayed a significant interaction with sex, i.e. higher regression slope in either group.

### 3.4 SBM results: sex differences

Eight components/networks showed significant sex difference (**Figures 2B, D**). Higher loading-coefficients in females than males were observed in components involving the amygdala alongside (para)hippocampal cortex (component 18), temporal pole (18 and 28), sensorimotor cortex (19, 21 and 31), right temporo-parietal junction (29), inferior and superior parietal lobuli (21, 29 and 31), alongside cerebellum (7, 19 and 28) (**Figure 2B**). Higher loading-coefficients in males than females were observed in the medial occipital cortex (lingual gyrus, calcarine gyrus and cuneus; component 14) (**Figure 2D**).

### 3.5 SBM results: correlation with BEES and IRI (sub)scores

Multiple regression models unveiled the contribution of specific structural networks to specific empathy subscores. The amount of variance explained (R^2^) by the overall models ranged from 0.26 to 0.45, and the R^2^ of each structural network from 0.15 to 0.29. All the variance inflation factors (VIFs) were beyond our maximum threshold of 4 (range: 1.17-1.42), thus excluding issues of multicollinearity among predictors. Some networks displayed significantly larger loading coefficients in females than males, while none of them was more represented in males than females.

The “Emotional empathy” BEES score was significantly associated with GMV in bilateral amygdala, hippocampus, parahippocampal gyrus and temporal pole (component 18, female dominant; *R* ^2^ = 0.20, *p*=0.002), bilateral sensorimotor cortex and superior parietal lobule (component 21, female dominant; *R* ^2^ = 0.16, *p*=0.037), right postcentral gyrus and bilateral cerebellum (component 19, female dominant; R^2^ = 0.16, p=0.016) and medial superior frontal gyrus (component 17; *R* ^2^ = 0.15, *p*=0.047) (model *R* ^2^ = 0.26, *p*=0.000001; **Supplementary Table S5**; **Figures 3B, C**). IRI Personal distress reflected in GMV in hippocampus and parahippocampal cortex, extending into the amygdala bilaterally but with a right-hemispheric dominance (component 5; *R* ^2^ = 0.26, *p*=0.022), and in the bilateral inferior and superior parietal cortex extending into the postcentral gyrus (component 31, female dominant; *R* ^2^ = 0.29, *p*=0.039) (model *R* ^2^ = 0.41, *p*=0.000249; **Supplementary Table S6a**; **Figure 3E**). Empathic concern was significantly associated with GMV in the pars orbitalis of the inferior frontal gyrus and temporal pole bilaterally, right temporo-parietal junction (component 32; *R* ^2^ = 0.26, *p*=0.035), and right postcentral gyrus alongside bilateral cerebellum (component 19, female dominant; *R* ^2^ = 0.23, *p*=0.0008) (model *R* ^2^ = 0.45, *p*=0.000023; **Supplementary Table S6b**; **Figure 3G**). The structural networks associated with BEES and Personal distress commonly involved the amygdala, but the former extended rostrally towards the temporal pole (component 18) while the latter recruited the hippocampus and parahippocampal cortex (component 5). None of the retained SBM components explained a significant proportion of variability in Fantasy or Perspective-taking IRI subscores (model *p*=0.23 and 0.87, respectively). None of these analyses displayed a significant interaction with sex.

## 4 Discussion

We report novel morphometric evidence of individual and sex differences in specific empathy facets, showing that only emotional empathy subscores reflected in GM variations within well-defined *structural networks*.

Individual differences in *personal distress* reflected in GMV in a cluster encompassing the right medial temporal pole, amygdala, and the posterior-to-anterior ventral insula. Prior studies suggested that these structures represent both one’s own and others’ negative emotional experiences (64–66), thus potentially underpinning an inner simulation of aversive states (67), thereby enabling their affective sharing (68). In line with a simulationist view, a relationship with neurostructural variability in limbic/somatosensory structures might explain the “defensive” motivational consequences of the most automatic and self-oriented forms of empathy (69). The responsiveness of these regions might indeed mediate the “egoistic” motivation to avoid/withdrawing from the stressor to reduce the aversive arousal associated with personal distress (70), thereby hampering prosocial behaviors. The observed positive relationship between personal distress and anterior insular GMV confirms previous related evidence (39), while previous opposite findings (40) might reflect remodeling processes involving pruning and myelination (71, 72). Moreover, positive and negative correlations between personal distress and insular GMV resulted from studies performed in Western (39) and Eastern (32, 40) contexts, respectively, which highlights cultural background as a potential modulating factor deserving consideration in future studies (73).

Unlike personal distress, the other-oriented emotional reaction of *empathic concern* increases one’s motivation towards prosocial behavior (74) through self-other distinction processes enabling a safer approach to another’s distress (70). This hypothesis fits with the present VBM and SBM evidence that this empathy trait correlates with GMV in the medial and sensorimotor/inferior parietal cortex, respectively. These areas might jointly underpin key processes for empathic concern, such as affective arousal through the sensorimotor cortex (75), vicariously experienced in third-person via the role of the medial and inferior parietal cortex in agency (76, 77) and top-down attentional control (78–80). These processes might support the perspective/attentional shift enabling empathic concern without personal distress (81), thereby explaining the role of parietal areas in concern for others in need and, more generally, in prosocial behavior (82, 83). While empathic concern was associated with the medial precuneus in VBM results, GMV in this region also reflected individual differences in the IRI Fantasy score. Rather than a structural network associated with specific empathic traits, the medial precuneus may therefore support unspecific visual imagery processes (84, 85) subserving different empathy facets. Such general-purpose contribution to empathic reactions might involve the tendency to imaginatively transpose oneself into fictional situations (i.e., Fantasy; 86) or to imaginatively represent positive social interactions (i.e., Empathic concern; 87, 88).

While this hypothesis on the putative role of the medial precuneus in empathy requires further inquiry, the lack of significant results for perspective-taking fits with the absence of structural networks specifically supporting the cognitive facets of empathy. The latter consideration may reflect the present behavioral and neurostructural evidence of qualitative differences between sharing others’ emotional experiences and representing their perspectives, paralleling their distinct phylogenetic and ontogenetic developmental trajectories. The ability to perceive and share others’ emotional states, crucial for parental care, pair-bonding and attachment (89, 90), also in non-human animals (91, 92), is structurally embodied in limbic networks which, since early infancy, are biologically hardwired to resonate with others’ situations. A basic self-other distinction develops from the second year of life (93), when vicarious reactions of personal distress are gradually replaced by empathic concern (94). Instead, some features of mentalizing and perspective-taking have evolved uniquely in humans (95), and their late development (96, 97) mirrors the maturation of complex brain networks, which may reflect in specific activations during socio-cognitive task (5), but not in clear-cut neurostructural substrates. The lack of structural correlates for perspective-taking fits with previous results (39), showing that perspective-taking is (negatively) associated with insular activity during pain perception, and not with GMV (32).

These qualitative differences across emotional and cognitive empathy in turn relate to well-known sex differences, i.e., females’ higher responsiveness to others’ emotional states, possibly reflecting their prominence as caregivers (98, 99), which can emerge either from an evolutionary pressure (100) or cultural expectation (101). Consistently with both this view and previous evidence (48), females scored higher than males in emotional empathy (BEES, personal distress and empathic concern). Morphometric analyses allowed to ground these behavioral observations of females’ superior emotional empathy in increased limbic and somatosensory GMV, possibly supporting the stronger females’ disposition to understand and share others’ emotional states (43).

Supporting multicomponential empathy models (7), our results highlight a clear distinction, at the neurostructural level, between empathic concern and personal distress. Despite largely overlapping findings from VBM and SBM, only the former approach highlighted the involvement of the insula, alongside an association between precuneus volume and both Fantasy and Empathic concern, while the potential contribution of cerebellum was uniquely shown by SBM. Considering the importance of these regions for social cognition (23, 102), these findings suggest that both VBM and SBM should be used to investigate its neurostructural underpinnings (45). Moreover, the absence of a neurostructural basis for perspective-taking, previously associated with a functional brain network (6), suggests that its putative association with empathic concern should be rather investigated with brain activation paradigms (8).

There are limitations to our findings. While using both the BEES and four IRI subscores increases data robustness (19, 21), concerns have been raised about the psychometric validity of the latter scale (52; but see 54). Moreover, since self-report questionnaires require emotional insight and a willingness to disclose personal information (21) and might be biased by social desirability (52), their outcomes should be supported by behavioural task performance (103).

Notwithstanding these limitations, our findings ground a multicomponential view of empathy in specific neurostructural clusters or networks, representing a novel reference on the differential strength of emotional vs. cognitive empathy in normal conditions, and their breakdown in neuropsychiatric conditions such as autism (e.g., 104), Parkinson’s disease (105) and fronto-temporal dementia (106). Our results might guide the design of innovative treatments for enhancing empathic skills, including social skills training (107) and neuromodulation (108), as well as the assessment of their effectiveness at the neural level through randomized controlled trials.

## Data Availability

The data that support the findings of this study are available from the corresponding author upon reasonable request.
