# Supplementary material for: The neurostructural bases of empathy: morphometric evidence for a multicomponential approach
Source: Front Psychiatry. 2025 Apr 10;16:1544632. doi: 10.3389/fpsyt.2025.1544632 (PMC12018333; doi:10.3389/fpsyt.2025.1544632)
Supplement: Supplementary file 1 [file DataSheet1.pdf]

Supplementary materials

Supplementary Table 1. Descriptive statistics

	BEES	IRI tot	IRI PD	IRI EC	IRI FA	IRI PT
**Females**						
Mean	37.781	94.766	18.078	26.813	24.625	25.250
SD	19.769	10.291	4.233	3.707	4.413	4.032
**Males**						
Mean	16.350	86.733	15.633	23.617	22.867	24.617
SD	19.541	9.630	3.687	3.189	4.504	4.009
**2 sample t-test**						
t-value	6.067	4.480	3.420	5.131	2.195	0.877
p-value	<0.001	<0.001	<0.001	<0.001	0.030	0.382

Supplementary Table 1. The table reports the mean and standard deviation (SD) of BEES score, IRI total score, as well as IRI Personal distress (PD), Empathic concern (EC), Fantasy (FA) and Perspective-taking (PT) sub-scores, separately for female and males participants, alongside the statistical significance of sex differences. Red font denotes a significant correlation (p<0.05).

Supplementary Table 2. Correlation table

		**BEES**	**IRI tot**	**IRI PD**	**IRI EC**	**IRI FA**	**IRI PT**
**BEES**	**Correlation index**		**0.711**	**0.515**	**0.673**	**0.475**	**0.192**
	**p-value**		***p*<0.01**	***p*<.001**	***p*<.001**	***p*<.001**	***p*=.033**
**IRI tot**	**Correlation index**	**0.711**		**0.653**	**0.724**	**0.678**	**0.545**
	**p-value**	***p*<0.01**		***p*<.001**	***p*<.001**	***p*<.001**	***p*<.001**
**IRI PD**	**Correlation index**	**0.515**	**0.653**		**0.343**	**0.305**	**0.041**
	**p-value**	***p*<.001**	***p*<.001**		***p*<.001**	***p*=.001**	***p*=.650**
**IRI EC**	**Correlation index**	**0.673**	**0.724**	**0.343**		**0.282**	**0.313**
	**p-value**	***p*<.001**	***p*<.001**	***p*<.001**		***p*=.002**	***p*<.001**
**IRI FA**	**Correlation index**	**0.475**	**0.678**	**0.305**	**0.282**		**0.102**
	**p-value**	***p*<.001**	***p*<.001**	***p*=.001**	***p*=.002**		***p*=.259**
**IRI PT**	**Correlation index**	**0.192**	**0.545**	**0.041**	**0.313**	**0.102**	
	**p-value**	***p*=.033**	***p*<.001**	***p*=.650**	***p*<.001**	***p*=.259**	

Supplementary Table 2. The table reports Pearson's correlation indexes, and the respective p-values, among BEES score, IRI total score, as well as IRI Personal distress (PD), Empathic concern (EC), Fantasy (FA) and Perspective-taking (PT) sub-scores. Red font denotes a significant correlation (p<0.05).

Supplementary Table 3. VBM Gender differences

Cluster-size	Hemisphere	Anatomical region		x	y	z	t-value
		**A. Females > Males**					
9248	Left	Inferior Frontal Gyrus (pars Opercularis)	Area 44	-57	12	18	5.76
	Left	Inferior Frontal Gyrus (pars Orbitalis)		-38	45	-12	4.93
	Left	Middle Orbital Gyrus		-28	44	-12	4.59
	Left	Superior Orbital Gyrus		-14	38	-20	4.57
	Left	Insula Lobe		-42	12	-5	5.17
	Left	Insula Lobe		-40	-4	-2	5.13
	Left	Insula Lobe	Insula (Id1)	-44	-19	-3	5.37
	Left	Hippocampus	Hipp (CA)	-28	-16	-17	4.72
8932	Right	Insula Lobe		46	3	1	6.13
	Right	Insula Lobe		42	16	-5	5.11
	Right	Amygdala		26	4	-18	5.81
	Right	Amygdala	Amyg (LB)	34	-3	-23	5.16
	Right	Amygdala	Amyg (LB)	33	0	-26	5.1
	Right	Hippocampus	Hipp (CA)	38	-28	-15	4.71
	Right	Superior Temporal Gyrus		45	-13	-5	5.59
	Right	Middle Temporal Gyrus		56	-36	-6	4.99
935	Left	Supplementary Motor Area		-2	24	63	5.58
	Left	Superior Medial Gyrus		-8	35	60	4.69
	Right	Superior Medial Gyrus		3	51	45	4.14

1624	Right	Anterior Cingulate Cortex		4	33	25	5.17
	Right	Caudate Nucleus		12	12	3	4.61
1039	Left	Postcentral Gyrus	Area 4a	-32	-30	66	4.41
	Left	Precentral Gyrus	Area 6	-39	-16	58	4.14
	Left	Postcentral Gyrus	Area 1	-32	-37	69	3.96
		**B. Males > Females**					
1867	Left	Cuneus		3	-94	22	6.94

Supplementary Table 3. From left to right, the table reports the extent (in number of 1.5 x 1.5 x 1.5 mm3 voxels), hemispheric lateralization, anatomical labeling based on the Anatomy Toolbox (v2.2c; Eickhoff et al., 2005), stereotactic coordinates and statistical-value of the brain structures in which grey matter (GM) volume was significantly larger in females than males (top) or vice versa (bottom) in VBM analyses ($p<0.05$ corrected for multiple comparisons). Id1: insular dysgranular sector; CA: Hippocampus Cornu Ammonis; LB: LateroBasal amygdala nuclei

Supplementary Table 4. VBM results: BEES and IRI scores

Cluster-size	Hemisphere	Anatomical region		x	y	z	t-value
		**A. BEES**					
3860	Left	Middle Temporal Gyrus		-51	-7	-24	4.58
	Left	Medial Temporal Pole		-40	23	-38	4.32
	Left	Inferior Temporal Gyrus		-52	-7	-33	4.09
2002	Left	Hippocampus	Amyg (LB)	-32	-9	-17	4.92
	Left	Fusiform Gyrus	Hipp (CA)	-36	-18	-24	2.9
	Left	ParaHippocampal Gyrus	Hipp (SUB)	-24	-30	-15	2.76
1948	Right	Medial Temporal Pole		39	24	-35	4.07
	Right	Temporal Pole		45	20	-27	3.87
	Right	Amygdala	Amyg (LB)	36	-1	-24	2.88
	Right	Hippocampus	Hipp (CA)	28	-12	-20	2.85
	Right	ParaHippocampal Gyrus	Hipp (CA)	24	-10	-23	2.83
		**B. IRI Personal distress**					
3008	Right	Right Temporal Pole		33	8	-21	4.06
	Right	Right Putamen		32	-6	0	3.84
	Right	Right Insula Lobe		26	24	-12	2.55
		**C. IRI Empathic concern**					
2186	Left	Middle Cingulate Cortex		-9	-30	33	4.26
	Left	Precuneus	SPL (5M)	-9	-49	54	3.46
	Right	Middle Cingulate Cortex		10	-31	34	3.3
	Right	Precuneus	SPL (5M)	10	-48	55	3.18
872	Right	Middle Occipital Gyrus		40	-81	34	4.33

	Right	Angular Gyrus (TPJ)	SPL (7A)	32	-66	49	2.93
		**D. IRI Fantasy**					
3037	Left	Precuneus	Area 3a	-9	-49	61	4.38
	Left	Precuneus	SPL (5M)	-6	-48	64	4.36
	Left	Precuneus	SPL (7A)	-9	-58	66	4
	Left	Postcentral Gyrus	Area 1	-24	-39	70	3.79
	Right	Superior Parietal Lobule	SPL (7A)	16	-61	58	3.66
	Right	Precuneus	SPL (5M)	3	-52	54	3.56
	Right	Precuneus	SPL (7A)	8	-60	58	2.68

Supplementary Table 4. From left to right, the table reports the extent (in number of 1.5 x 1.5 x 1.5 mm^3 voxels), hemispheric lateralization, anatomical labeling based on the Anatomy Toolbox (v2.2c; Eickhoff et al., 2005), stereotactic coordinates and statistical-value of the brain structures in which GM volume was significantly positively correlated with the BEES "emotional empathy" score or with the different IRI subscores in VBM analyses ($p<0.05$ corrected for multiple comparisons). LB: LateroBasal amygdala nuclei; CA: Hippocampus Cornu Ammonis;. SUB: Hippocampus Subiculum; SPL: Superior Parietal Lobule.

Supplementary Table 5. SBM results: BEES emotional empathy

Cluster-size	Hemisphere	Anatomical region		x	y	z	t-value
		**Component 18, BEES**					
10846	Left	Inferior Temporal Gyrus	Hipp (EC)	-27	0	-41	9.66
	Left	Middle Temporal Gyrus		-51	-16	-11	2.66
9543	Right	Fusiform Gyrus	Hipp (EC)	24	6	-41	8.2
	Right	ParaHippocampal Gyrus	Hipp (EC)	28	-7	-36	7.72
	Right	Inferior Temporal Gyrus		57	-14	-29	5.98
357	Left	Middle Temporal Gyrus		-42	-63	15	4.7
		**Component 19, BEES, Empathic concern**					
4034	Left	Cerebellum	Lobule VIIa Crus II	-39	-69	-44	9.21
3460	Right	Cerebellum	Lobule VIIa Crus II	38	-75	-45	9.01
846	Right	Postcentral Gyrus	Area 1	26	-42	68	4.67
	Right	Postcentral Gyrus	Area 2	32	-36	51	3.09
	Right	Paracentral Lobule	Area 4a	10	-37	76	2.53
534	Left	Cerebellum	Lobule IX	-2	-58	-54	5.24
		**Component 21, BEES**					
20374	Right	Paracentral Lobule	Area 4a	0	-30	69	8.28
	Left	Precentral Gyrus	Area 6	-28	-24	66	6.19
	Right	Superior Frontal Gyrus	Area 6	22	-7	63	5.29
	Right	Superior Parietal Lobule	SPL (7PC)	22	-52	64	4.96
	Right	Precentral Gyrus	Area 6	22	-18	72	4.86
	Left	Superior Parietal Lobule	SPL (7A)	-20	-51	61	4.64
	Right	Precentral Gyrus	Area 6	30	-18	69	4.62

	Right	Postcentral Gyrus	Area 1	40	-30	63	3.47
	Left	Precuneus		-4	-58	52	2.67
	Left	Postcentral Gyrus	Area 1	-52	-22	51	2.67
1344	Right	Thalamus	Th-Temporal	2	-15	7	4.23
	Left	Thalamus	Th-Temporal	-9	-18	13	3.7
484	Right	IFG (p. Triangularis)	Area 45	51	21	15	4.61
		**Component 17, BEES**					
597	Left	Left Precentral Gyrus	Area 6	-38	-12	61	4.29
425	Left	Left Superior Frontal Gyrus		-15	24	55	3.51
413	Right	Right Superior Temporal Gyrus		52	-39	9	3.37
320	Left	Left Inferior Temporal Gyrus		-57	-30	-18	3.83

Supplementary Table 5. From left to right, the table reports the extent (in number of 1.5 x 1.5 x 1.5 mm^3 voxels), hemispheric lateralization, anatomical labeling based on the Anatomy Toolbox (v2.2c; Eickhoff et al., 2005), stereotactic coordinates and statistical-value of the brain structures in which the loading coefficient of GM structural networks highlighted by SBM analyses was significantly positively correlated with the BEES “emotional empathy” score ($p<0.05$ corrected for multiple comparisons). EC: Hippocampus Enthorinal Cortex; SPL: Superior Parietal Lobule.

Supplementary Table 6. SBM results: IRI emotional empathy

Cluster-size	Hemisphere	Anatomical region		x	y	z	t-value
		**A: Component 5, Personal distress**					
3184	Right	ParaHippocampal Gyrus	Hipp (CA)	32	-28	-15	7.2
	Right	ParaHippocampal Gyrus	Hipp (EC)	21	-3	-29	3.61
2567	Left	Hippocampus	Hipp (CA)	-30	-30	-14	7.36
	Left	Fusiform Gyrus	Amyg (LB)	-30	-7	-32	4.73
		**Component 31, Personal distress**					
3701	Left	Inferior Parietal Lobule		-32	-51	39	13.49
	Left	Superior Parietal Lobule	SPL (7PC)	-36	-49	60	6.25
	Left	Precuneus	SPL (7A)	-12	-55	57	3.27
3609	Right	Inferior Parietal Lobule	hIP3	33	-49	42	13.29
	Right	Superior Parietal Lobule	Area 2	36	-45	62	5.88
	Right	Precuneus		8	-55	49	2.89
	Right	Superior Parietal Lobule	SPL (7A)	18	-61	58	2.68
		**B. Component 32, Empathic concern**					
2603	Right	Inferior Frontal Gyrus (p. Orbitalis)		22	14	-23	5.42
	Left	Inferior Frontal Gyrus (p. Orbitalis)		-24	14	-24	5.62
1912	Left	Cerebellum	Lobule VIIa Crus I	-45	-49	-36	10.78
1774	Right	Cerebellum	Lobule VIIa Crus I	45	-50	-36	9.2
1630	Left	Middle Temporal Gyrus		-42	-61	13	7.52
	Left	Middle Occipital Gyrus		-44	-73	24	4.49
813	Right	Inferior Parietal Lobule	hIP3	34	-45	40	6.76
		**Component 19, Empathic concern, BEES**					
4034	Left	Cerebellum	Lobule VIIa Crus II	-39	-69	-44	9.21
3460	Right	Cerebellum	Lobule VIIa Crus II	38	-75	-45	9.01
846	Right	Postcentral Gyrus	Area 1	26	-42	68	4.67

	Right	Postcentral Gyrus	Area 2	32	-36	51	3.09
	Right	Paracentral Lobule	Area 4a	10	-37	76	2.53
534	Left	Cerebellum	Lobule IX	-2	-58	-54	5.24

Supplementary Table 6. From left to right, the table reports the extent (in number of 1.5 x 1.5 x 1.5 mm^3 voxels), hemispheric lateralization, anatomical labeling based on the Anatomy Toolbox (v2.2c; Eickhoff et al., 2005), stereotactic coordinates and statistical-value of the brain structures in which the loading coefficient of GM structural networks highlighted by SBM analyses was significantly positively correlated with the IRI "emotional empathy" subscores of personal distress and empathic concern ($p<0.05$ corrected for multiple comparisons). CA: Hippocampus Cornu Ammonis; EC: Hippocampus Enthorinal Cortex; LB: LateroBasal amygdala nuclei; SPL: Superior Parietal Lobule; hIP: human IntraParietal.
